# Influence of Major Polyphenols on the Anti-*Candida* Activity of *Eugenia uniflora* Leaves: Isolation, LC-ESI-HRMS/MS Characterization and In Vitro Evaluation

**DOI:** 10.3390/molecules29122761

**Published:** 2024-06-10

**Authors:** Camylla Janiele Lucas Tenório, Thainá dos Santos Dantas, Lucas Silva Abreu, Magda Rhayanny Assunção Ferreira, Luiz Alberto Lira Soares

**Affiliations:** 1 Laboratory of Pharmacognosy, Department of Pharmaceutical Sciences, Federal University of Pernambuco, Recife 50670-901, PE, Brazil; camylla.tenorio@ufpe.br (C.J.L.T.); thaina.dantas@ufpe.br (T.d.S.D.); magda.raferreira@ufpe.br (M.R.A.F.); 2 Post-Graduate Program in Pharmaceutical Sciences, Federal University of Pernambuco, Recife 50670-901, PE, Brazil; 3 Post-Graduate Program in Therapeutic Innovation, Federal University of Pernambuco, Recife 50670-901, PE, Brazil; 4 Chemistry Institute, Fluminense Federal University, Niterói 24020-150, RJ, Brazil; abreu_lucas@id.uff.br; 5 Pharmaceutical Abilities Laboratory, Pharmacy, School of Health and Life Sciences, Catholic University of Pernambuco, Recife 50050-900, PE, Brazil

**Keywords:** flavonoids, tannins, purification, *Candida* spp.

## Abstract

The content of chemical constituents in *Eugenia uniflora* leaf extracts correlates positively with biological activities. The experimental objective was to carry out the phytochemical screening and purification of the major polyphenols from the leaves of *E. uniflora*. In addition, the anti-*Candida* activity of the hydroalcoholic extract, fraction, subfractions and polyphenols purified were evaluated. After partitioning of the extract with ethyl acetate, the fractions were chromatographed on Sephadex^®^ LH-20 gel followed by RP-flash chromatography and monitored by TLC and RP-HPLC. The samples were characterized by mass spectrometry (LC-ESI-QTOF-MS^2^) and subjected to the microdilution method in 96-well plates against strains of *C. albicans*, *C. auris,* and *C. glabrata*. Myricitrin (93.89%; *w*/*w*; *m*/*z* 463.0876), gallic acid (99.9%; *w*/*w*; *m*/*z* 169.0142), and ellagic acid (94.2%; *w*/*w*; *m*/*z* 300.9988) were recovered. The polyphenolic fraction (62.67% (*w*/*w*) myricitrin) and the ellagic fraction (67.86% (*w*/*w*) ellagic acid) showed the best antifungal performance (MIC between 62.50 and 500 μg/mL), suggesting an association between the majority constituents and the antifungal response of *E. uniflora* derivatives. However, there is a clear dependence on the presence of the complex chemical mixture. In conclusion, chromatographic strategies were effectively employed to recover the major polyphenols from the leaves of the species.

## 1. Introduction

Chromatography is a physical, chemical, and/or mechanical separation method based on the interactions and distribution of the components of a sample between two immiscible phases. It has become a very effective and ubiquitous technique in various areas for analytical and preparatory purposes, the latter being the main general separation strategy for the purification and recovery of chemical species, among them polyphenols, one of the largest groups of substances distributed in the plant kingdom [1,2].

However, the recovery of polyphenols from herbal species remains a challenge due to the complex multi-component constitution of these biological matrices. Most of the tests consider the development and application of combined methodologies within the world of chromatography [3,4]. The recovery of these constituents is desirable for the structural and pharmacological elucidation of bioactives, contributing to the collection of information available for this class of metabolites [5,6].

Polyphenols, in general, are widely recognized for their therapeutic properties in plant species [7,8]. Gallic and ellagic acids, together with myricitrin, are examples of polyphenolic structures that stand out as the main constituents of *Eugenia uniflora* Linn leaves. Similar to other species, the concentration of these components in the extracts and fractions exhibited a positive correlation with the biological activities described for the plant, particularly regarding antifungal activity against strains of *Candida* spp. through various mechanisms [9,10].

*Eugenia uniflora* belongs to one of the largest genera in the Myrtaceae family and holds significant socio-economic potential due to the commercial exploitation of its plant parts and its therapeutic use in traditional medicine [11,12]. Although native to Brazil, Eugenia uniflora is also commonly found in other South American countries and is remarkably adaptable [13], positioning the species as a potential plant source to address the increasing demand for new therapeutic agents derived from plants.

The growing interest in this species is evidenced by the increase in the number of publications in recent years. Publications using the leaves focus on in vitro and/or in vivo research into the pharmacological properties of its extracts and fractions [14,15,16]; evaluation of their toxicological safety [17,18]; development of efficient methodologies and strategies for obtaining, identifying, characterizing, and quantifying their constituents [14,19,20,21]. In addition, there are reports on the development of intermediate products and pharmaceutical formulations containing leaf extracts of the species [22,23].

In this context, it is crucial to identify, develop, evaluate, and optimize separation strategies for the main secondary metabolism compounds present in *E. uniflora* leaves. This will ensure the availability of these substances, enabling both individual and combined pharmacological investigations as well as monitoring their presence in different matrices of the extract and its fractions. This approach not only meets the need for a wide range of research possibilities on the constituents of the species but also presents itself as a viable alternative to commercialized reference standards.

## 2. Results

### 2.1. Spray-Dried Crude Extract and Enriched Fraction

To produce a total of 810 g of Spray-dried Crude Extract (SDCE), approximately 9.36 kg of herbal material is required, assuming the same extraction and drying processes described in this study are used. Given the significantly large amount needed, the process was evaluated in triplicate, and the obtained yields were calculated (Table 1). The Ethyl Acetate Fraction (EAF) constituted about 2.27% (*w*/*w*) of the SDCE.

Analysis of the samples using High-Performance Liquid Chromatography with a diode array detector (HPLC-DAD) enabled the identification of the predominant peaks in the SDCE, corresponding to Gallic Acid (GA) at 7.6 min, myricitrin (MyR) at 22.3 min, and Ellagic Acid (EA) at 23.9 min (Figure S1A) [19]. GA exhibited absorption maxima at 214 nm and 271 nm, which corroborates literature reports that describe maxima ranging between 214 and 215 nm and between 271 to 273 nm. GA, along with its various polymeric derivatives (gallotannins), whether glycosylated or substituted, is extensively documented in species of the *Eugenia* spp. genus [14,21]. For MyR, absorption maxima were observed at 207 nm, 259 nm, and 353 nm, which are characteristic of flavonols. This is consistent with literature descriptions of absorption maxima ranging between 207 and 212 nm, 259 and 260 nm, and 352 and 358 nm [19,24]. For EA, absorbance maxima were noted at 253 nm and 367 nm, aligning with literature findings that describe absorption maxima between 253 and 255 nm and 360 and 368 nm. Additionally, this constituent is also frequently reported in the *Eugenia* spp. genus [19,25,26].

Comparing the results of SDCE and EAF, both evaluated at 1 mg/mL, the major polyphenols in EAF are up to 20 times more concentrated than in SDCE. While GA, MyR, and EA show peak areas of 3.44, 9.58, and 2.38 mAU in SDCE, respectively, peak areas of 34.31, 78.17, and 45.51 mAU were found in EAF (Figure S1B). Despite the yield of 2.27 ± 0.09% (3.97% *w*/*w*), the Liquid–Liquid Extraction (LLE) strategy efficiently concentrated the three major constituents from the leaves of the species. Enriched fractions are preferred samples for application in polyphenol recovery methodologies in herbal matrices [2,27]. When applied as an initial step, LLE reduces the excess of apolar interferents present in the complex matrix, and, consequently, with the removal of undesirable components, the compounds of interest are concentrated at the end of the process. In general, the isolation of polyphenols from enriched fractions, compared to the direct use of crude extracts, shows better separation and recovery efficiency [2,27,28].

### 2.2. Screening Tests for Subfractionation

#### 2.2.1. EAF Processing by Reversed-Phase Flash Chromatography (RP-FC)

**Figure 1:**
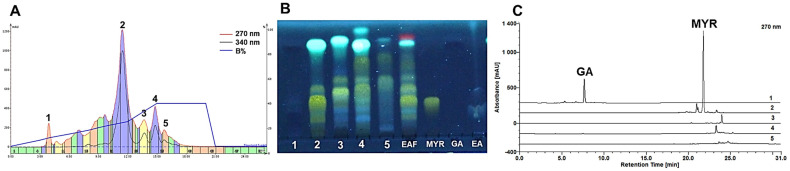
Chromatogram of test 2 of the RP-FC screening (**A**) and TLC of the five collected sub-fractions derivatized with 5% (*w*/*v*) AlCl_3_ (**B**). Chromatograms of the sub-fractions from test 2 by HPLC at 270 nm (**C**).

The optimization of methodologies and conditions was a critical step in devising a successful strategy. According to the literature, the Reversed-Phase Flash Chromatography (RP-FC) system is effectively used for the analysis and separation of polyphenolic structures, facilitating the separation of a broad spectrum of solutes with medium to high polarity that exhibit diverse interactions with the octadecylsilane of the stationary phase [5,29]. When the technique was applied with the transposition of the analytical method, no effective chromatographic separation of any individual polyphenol in the EAF was observed (Figure S2A). Prolonging the run time enhanced the chromatographic profile (Figure 1A), and GA was semi-purified in subfraction 1, showing the same retention factor (RF) as the standard (0.65) when monitored by Thin Layer Chromatography (TLC) (Figure 1B), which was confirmed by HPLC-DAD, evaluated at 1 mg/mL, at 7.6 min (Figure 1C). A total of 10.20 mg of semi-purified GA was recovered, amounting to 5.09% (*w*/*w*) of the utilized EAF. The RP-FC method proved inefficient for the recovery of semi-purified or concentrated MyR and EA in a single subfraction. In complex samples, such as extracts and concentrated fractions, other matrix components, besides the targeted major polyphenols, interact with the stationary phase and/or possess characteristics incompatible with the UV-Vis detection system, potentially disrupting the separation process [30]. This screening indicated that the technique is more suitably applied to less complex subfractions, thereby reducing the number of fractions processed and affirming its efficient use in the final stages [18].

#### 2.2.2. EAF Processing by Size Exclusion Chromatography

**Figure 2:**
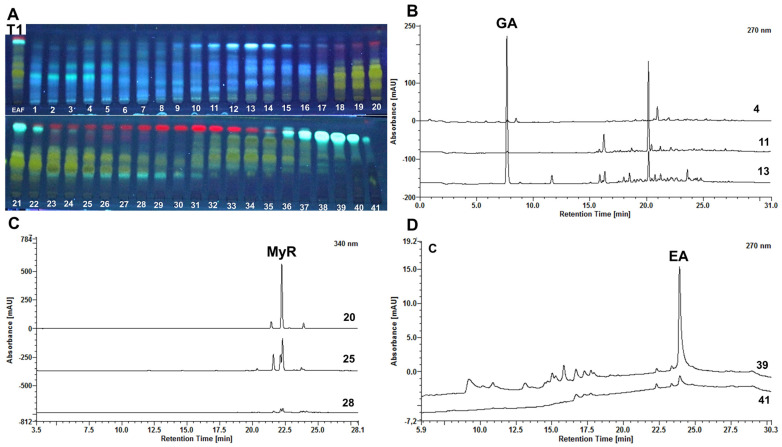
TLC of the subfractions collected in test 1 of the SEC screening derivatized with 5% (*w*/*v*) AlCl_3_ (**A**), Chromatograms of sub-fractions 4, 11, 13 at 270 nm (**B**), 20, 25, and 28 at 340 nm (**C**) and 39, 41 at 270 nm (**D**) from test 1 of the SEC screening.

In the initial Size Exclusion Chromatography (SEC) test (Test 1), 41 subfractions were obtained, with blue bands observed in subfractions 1–17, predominantly yellow bands in subfractions 18–35, and in subfraction 39, an apparently semi-purified fluorescent band. (Figure 2A). The results indicate an effective separation by metabolite class. Beyond size exclusion separation, the Sephadex^®^ LH-20 gel, a dextran cross-linked with hydroxypropylated spheres, is also notable for adsorption separation, creating a chromatographic medium with amphiphilic and adsorptive properties modulated by the mobile phase [31,32]. Dual separation is advantageous for the fractionation and isolation of polyphenolic constituents, accommodating a broad range of low- to medium-molecular-weight structures with varying polarities [33,34].

The two mechanisms acting concurrently in the system, retention by adsorption, are attributed to the formation of hydrogen bonds with the hydroxyl groups of the polyphenols. The affinity strength is directly proportional to the number of hydrogen atoms, which also correlates with the molecular weight of the compound. Consequently, the general rule of SEC, where elution occurs in reverse order to the size of the analyte, is modified in tests with Sephadex^®^ LH-20. In this experiment, simple phenolic constituents, despite their low molecular weight, eluted first, likely due to their interaction with the initially more aqueous medium [1,35].

During the tests, adjusting the mobile phase from higher to lower polarity using a gradient between water and methanol resulted in the sequential elution of phenolic acids, glycosylated flavonoids, flavonoid aglycones, and tannins, displaying blue, yellow, and fluorescent bands (Figure 2A), traits typical of polyphenolic components [36]. Fractions 4, 11, 13, 20, 25, 28, 39, and 41 were chosen for injection into the HPLC at 0.25 mg/mL. The selection criteria took into account the TLC profile of each fraction and the amount recovered that was viable for weighing and preparing the sample for analysis.

Hydroalcoholic mixtures with methanol (MeOH) were initially chosen based on methodologies found in the literature for the isolation of polyphenols by Sephadex^®^ LH-20 [31]. Ethanol (EtOH) is less widely reported but is also used [35]. In this sense, EtOH was tested as a comparative criterion (Test 2), and the separation process observed showed advantages over MeOH. The total elution time was 24 h, spread over 3 days with a flow rate of approximately 0.17 mL/min, while that of MeOH was 40 h, spread over 5 days with a flow rate of approximately 0.10 mL/min.

Reducing the procedure time is desirable since the aim of the study is to develop a methodology for optimized major polyphenol recovery. In this sense, the shorter the time, the more processes can be carried out in less time. Additionally, in terms of process costs, EtOH has a lower market price. When monitoring, it was possible to observe that the initial and final compounds were better separated (Figure S4). The volume of the bed and the exclusion limit vary depending on the solvent used in the process. For example, the gradual increase in EtOH not only has a greater capacity to reduce pore size when compared to MeOH but also subtly reduces the polarity of the bed [35,37].

In the SEC test with EtOH, GA was identified at 7.62 min in subfraction 16. The majority peak of the subfraction, despite the difference in retention time (20.19 min), resembles the GA spectrum and may be a derivative of GA (Figure S5A). In subfraction 20, MyR was identified at 22.23 min. Peaks 1 (21.42 min) and 3 (23.95 min) displayed spectra similar to MyR, suggesting the presence of other flavonoids (Figure S5B). In subfractions 40, 44, and 48, the spectra of the majority peaks were identified as ellagic acid at 23.84 min. Peaks 1 and 2, with retention times of 18.35 and 19.00 min, respectively, showed spectra similar to EA, a characteristic absorption of flavanols and flavanones [38].

Scaling the method from a smaller column to a larger one (Test 3) demonstrated reproducibility in the chromatographic profile, as monitored by TLC (Figure S6). A total of 64 sub-fractions were collected during the 38-h procedure, which was spread over 5 days with a flow rate of approximately 0.5 mL/min. The main objective of the study was to isolate the primary constituents of the species (GA, MyR, and EA) in sufficient quantities for other planned tests. To optimize the experimental time, the hydroethanolic ratio was adjusted, reducing the procedure time to 28 h, spread over 3.5 days, with a flow rate of approximately 0.54 mL/min. Sixty sub-fractions were collected (Figure S7), and the eluates containing MyR and GA started to be eluted from sub-fraction 18. The yields of the subfractions are described in Table S2.

### 2.3. Chromatographic Strategies for Major Polyphenol Recovery

#### 2.3.1. Fractionation by Sephadex^®^ LH-20

Based on the selection tests, SEC strategies were established for the initial processing of the EAF. Collections were made in volumes according to the profile of components observed during monitoring, reducing the time to 22 h, spread over 2 days, with 22 collections.

As in the tests, the eluate profiles were maintained, with the presence of blue and yellow bands [36] (Figure S8). Using the same elution profile, Tian, Liimatainen [39] reported similar results: simple phenolic acids were eluted in the first subfractions, followed by glycosylated flavonoids, flavones, and flavanones, and, lastly, tannins and ellagitannins, including EA. The authors also point out that the use of acetone as the final eluent is essential to reduce the adsorptive interaction of polymeric forms with the gel matrix [39], explaining why ellagitannins were eluted last in the system used.

Visual monitoring from SP1 to SP4 demonstrates the reproducibility of the chromatographic runs (Figure S8). Regarding the yields, in SP1, approximately 23.57% (*w*/*w*) of the yield corresponds to the MyR and GA-rich subfractions, and 2.7% (*w*/*w*) to the subsequent EA-rich subfractions (Table 2). The similar subfractions from all tests were combined according to their chromatograms, and the main subfractions were designated as the simple phenolics fraction (SPF), the main polyphenol fraction (MPF), and the last fractions (LF).

#### 2.3.2. Fractionation and Isolation of Major Polyphenols by Flash Chromatography

- Ellagic acid (EA)

**Figure 3:**
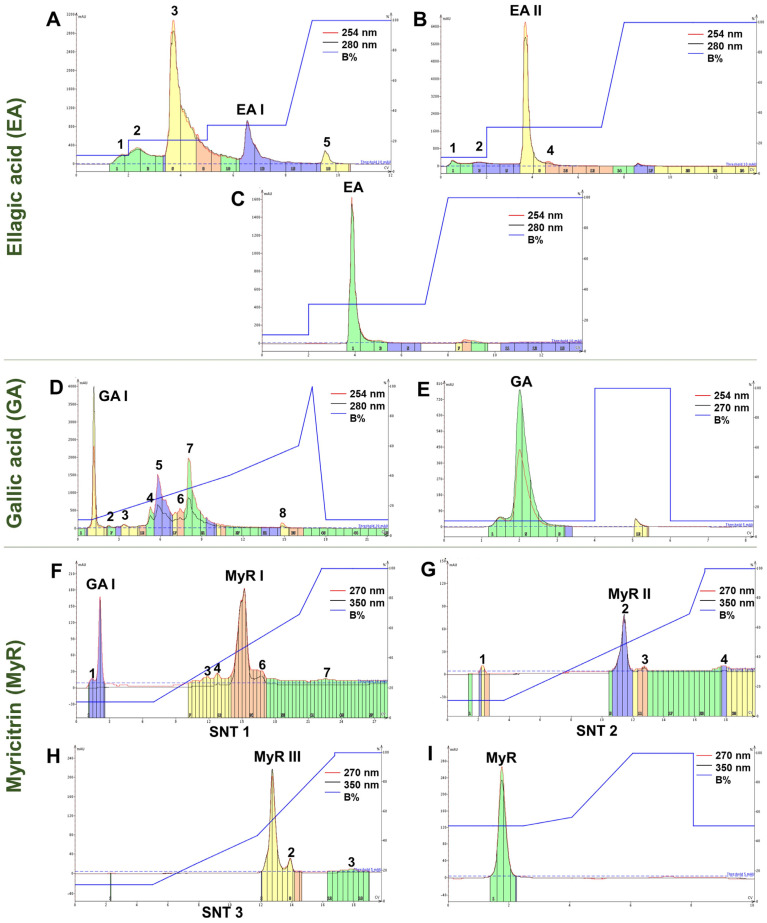
RP-FC chromatograms of LF subfractionation to obtain EA I (**A**), EA I to obtain EA II (**B**) and EA II to recover EA (**C**); MPF subfractionation (**D**) to obtain GA I (**D**) and GA I to recover GA (**E**); Subfrac-tionation of SNT 1 of MPF to obtain MyR I (**F**), SNT 2 to obtain MyR II (**G**), SNT 3 to obtain MyR III (**H**) and recovery of MyR from MyR III (**I**).

For the EA component, utilizing LF and the most optimized methodology, the peaks were partially separated (Figure 3A). Subfraction 4 (EA I), which corresponds to the semi-purified EA, was subsequently resubmitted to RP-FC (Figure 3B). From subfraction 3 (EA II), the purified EA was obtained (Figure 3C). The recovered substance was a fine, yellowish, odorless powder that was insoluble in water. EA can manifest as cream-colored needles or as a yellow powder with a water solubility of less than 1 mg/mL at 21 °C. It is odorless, weakly acidic, and incompatible with strong reducing agents [40].

In terms of yield, the yields of the EA-containing subfractions were low compared to the other major constituents, necessitating multiple processing steps to obtain optimal quantities of the purified product. The processing of LF yielded between 1.23 and 3.52% (*w*/*w*) of EA I, and 11.85% (*w*/*w*) of this was recovered as EA II (Table S2). Ultimately, the recovery of EA from EA II had a yield of 67.86% (*w*/*w*), with 1.90 mg collected from the processing of 2.8 mg of EA I.

- Gallic acid (GA)

The RP-FC methodology applied proved efficient in the semi-purification of GA from MPF. Peak 1 showed a band corresponding to GA on TLC (RF = 0.74) and was thus named GA I (Figure 3D). GA I was subjected to RP-FC, resulting in the recovery of GA (Figure 3E). The content recovered in GA I was brown, and after reprocessing the sample, an odorless, hygroscopic fine powder was obtained. This powder exhibited crystalline characteristics and was shaped like needles with a light brownish color. Purified GA is described as white or light brown needle-shaped crystals or powders [41]. The yields of GA I from MPF were up to 35.75% (*w*/*w*) (Table S3). From GA I, approximately 58.12% (*w*/*w*) of GA was recovered, totaling 29.12 mg.

- Myricitrin (MyR)

After processing the MPF using RP-FC in several chromatographic runs, it was observed that a portion of the sample, which was previously completely solubilized, precipitated at the start of elution. This resulted in the chromatographic profile and MyR recovery not being reproducible, leading to subsequent sample losses. Less concentrated samples were tested, and precipitation was observed again. As a result, an additional strategy of centrifugation was adopted. The chromatograms showed different chromatographic profiles for each processing of the supernatant (SNT), and the majority of peaks corresponded to MyR (Figure 3F–H).

During the drying process of the MyR I and II subfractions, an abundant light yellow powder and a darker colored powder were observed in the middle and at the edges of the flask, respectively. Monitoring by TLC revealed that separation still occurred during the evaporation of the solvent present in the eluate (Figure S9). A possible explanation is the saturation precipitation of MyR in MyR I and II, as well as its low solubility in aqueous media, given that the organic solvent evaporates more quickly. The precipitate is easily collected during drying. The powders collected in this process were also named MyR III.

The recovered powder was light yellow in color, had an unidentified characteristic smell, and was insoluble in water. MyR is characterized as a yellow powder that is practically insoluble in water, slightly soluble in ethanol, and has a characteristic bayberry aroma [42,43]. The recovery of MyR-rich fractions from MPF, including I and II, ranged from 50.52%, 61.60%, and 62.67% (*w*/*w*). The edges separated from the precipitate accounted for approximately 14.94% (*w*/*w*) of the total MyR I and II collected. Around 17.8 mg of MyR I yielded 10.23 mg (57.47% *w*/*w*) of MyR II (Table S4), and when this was subjected to RP-FC again, it showed yields of 89.32% (*w*/*w*) of MyR III and 93.89% (*w*/*w*) of purified MyR.

When the MPF was processed using this methodology, GA was also recovered, reserved, and named GA I. However, the yields of this fraction were subtly reduced when the detection range was changed: previously, in the recovery tests for GA, with detection set at 254 and 280 nm, the yields were 29.20% (*w*/*w*), while in these tests, with detection set at 270 and 350 nm, the yield was 20.47% (*w*/*w*).

### 2.4. Monitoring the RP-Flash Subfractionation and Isolation Process by HPLC-DAD

**Figure 4:**
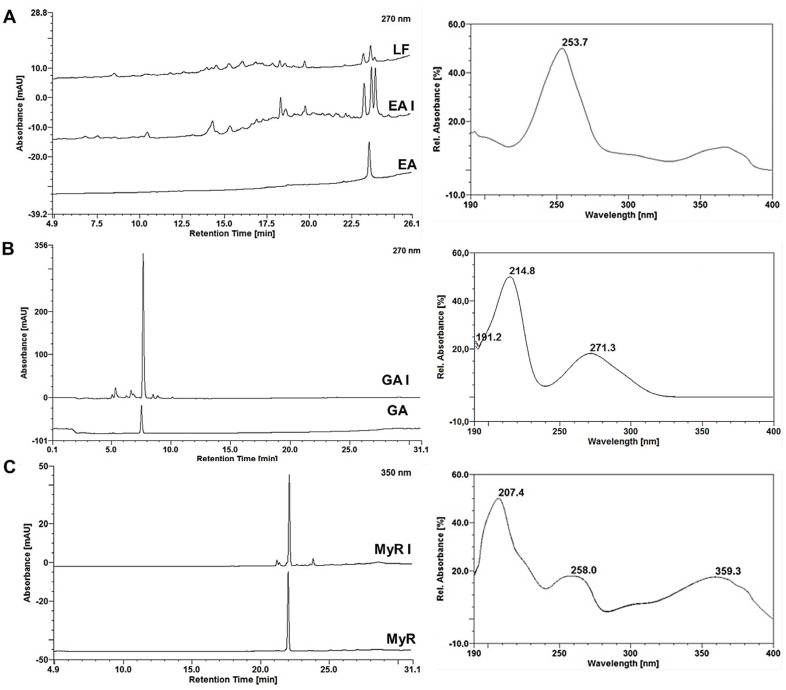
HPLC-DAD chromatograms of the Last Fraction (LF), Ellagic Acid after RP-LF processing of the last fraction (EA I) and purified Ellagic Acid at 270 (**A**); Gallic Acid I after RP-FC processing of the main polyphenols fraction (MPF) and purified Gallic Acid (GA) at 270 (**B**); Myricitrin after RP-FC pro-cessing of supernatant I of the MPF (MyR I) and purified Myricitrin (MyR) at 350 nm (**C**).

In the HPLC-DAD analysis, the purified EA showed a retention time of 23.52 min (Figure 4A) with a peak purity of 94.2% at 4.26 mAU. The absorption spectrum exhibits absorbance maxima at 253 and 367 nm [19,25,26]. The recovered GA displayed a retention time of 7.51 min (Figure 4B) and a peak purity of 99.90% at 4.93 mAU. Its absorption spectrum revealed absorption maxima at 214 and 271 nm [14,21]. As for the MyR recovered, it had a retention time of 21.99 min (Figure 4C), slightly reduced compared to that observed in MPF (23 min), and a peak purity of 99.9% with 4.49 mAU. The absorption spectrum recorded absorption maxima at 207, 259, and 353 nm [19,24]. Chromatograms obtained from the injection of LF at 250 μg/mL, EA I, GA I and MyR I at 25 μg/mL, and AE, AG, and MyR at 2 μg/mL.

### 2.5. Phytochemical Characterization of the Extract, Fractions, Subfractions and Isolates

#### 2.5.1. Phytochemical Profile

The 22 compounds were identified in *E. uniflora* leaf extract and fractions by the interpretation of their fragmentation patterns obtained from mass spectra (HRMS/MS experiments). Data provided by reference standards and the literature information was also employed for the comprehensive evaluation of samples. The retention times and mass spectrum data, along with peak assignments for compounds identified using negative ionization, are described in Table 3.

#### 2.5.2. Identification of Flavonols

The identification of these compounds was facilitated by the analysis of fragmentation pathways of ions in the negative modes and the observation that glycosidic residues (pentosyl (132 Da), rhamnosyl (146 Da), glucosyl (162 Da), and rutinosyl (308 Da) were cleaved sequentially and generated characteristic aglycone fragments compared to the available literature. Among these compounds, five compounds were identified as quercetin glycoside (16–20), eight were identified as myricetin glycosides (8, 12, 13), and among them the major constituent myricitrin (14) and two kaempferol glycosides (21 and 22) were identified. In addition, kaempferol, quercetin, and myricetin derivatives were observed on the basis of the main ion fragments produced on the MS/MS experiments, appearing at *m*/*z* 284 and 285 for kaempferol derivatives, *m*/*z* 300 and 301 for quercetin derivatives, and *m*/*z* 316 and 317 for myricetin derivatives; these pairs of ion fragments corresponded to the respective homolytic and heterolytic cleavage of the glycosidic bonds in these compounds [49]. Moreover, the ion fragments produced with *m*/*z* 179 and 151 were compared with literature data and attributed to the confirmation of the flavonol core (kaempferol, quercetin, or myricetin) (Table 3). All these compounds were identified previously in *E. uniflora* [15,44,45,46,48].

#### 2.5.3. Identification of Other Phenolic Compounds

Gallotannins were detected and were distinguished by their characteristic fragment ion spectra, yielding sequential losses of galloyl (*m*/*z* 152) and gallate (*m*/*z* 170) residues. The digalloyl-hexoside derivatives (4, 7, 9–11) were assinalated based on the [M−H]^−^ ion at *m*/*z* 483 or your derivatives and MS/MS produced as typical product ions at *m*/*z* 313, 271, 211, 193, 169, and 125 [15,44,45,46]. Additionally, the compounds citric acid (2), gallic acid (3), 5-*O*-Caffeoylquinic acid (5), 5-*O*-Coumaroylquinic acid (6) were identified based on [M−H]^−^ ions at *m*/*z* 191, 169, 353, 337, respectively. MS/MS data of these ions were compared with the literature data [15,44,45,46,47].

#### 2.5.4. Isolated Compounds from *E. uniflora*

Myricitrin, myricetin-*O*-rhamnoside, was identified with *m*/*z* of 463.0871 (1.07 ppm) and *m*/*z* 316.02 of its myricetin aglycone after glycosidic loss (Figure S6A). Its dimer was also found at *m*/*z* 927.1825 (−2.48 ppm). Peaks at *m*/*z* 151.0053, 178.9986, and 271.0234 are characteristic of flavonoid fragments, with the *m*/*z* 151 fragment being characteristic of the cleavage of the C ring [45]. Gallic acid was identified at *m*/*z* 169.0142 (6.5 ppm) (Figure S7B). In the same sample, its digallic acid dimer was identified at *m*/*z* 339.0358 (3.53 ppm) and *m*/*z* 483.1851 and 313.0577, corresponding to fragments of glycosylated diglycosyl derivatives [48]. Additionally, *m*/*z* 300.9986 was suggestive of ellagic acid (2.65 ppm). Ellagic acid itself was identified at *m*/*z* 300.9982 (1.01 ppm) (Figure S7C). The dimer at *m*/*z* 603.0044 (0.63 ppm) and the trimer at *m*/*z* 905.0109 (0.52 ppm) were also found in the same sample. Among the fragments described in the literature for ellagic acid, *m*/*z* of 183.0110, 249.0387, and 275.0167 were observed [50].

### 2.6. Antifungal Activity

The selection of *Candida* spp. strains for this study was based on the findings of an-tifungal tests previously published for the extract and fractions of *Eugenia uniflora*. The minimum inhibitory and fungicidal concentrations against *C. albicans* and *C. glabrata* showed promise [9,10]. It has also been observed that fractions enriched with the main constituents of the species exhibit better antifungal performance against strains of *Candida albicans* [9]. In this study, SDCE and EAF showed similar MIC (Minimum Inhibitory Concentration) and MFC (Minimum Fungicidal Concentration) values, which align with observations in the literature. Most of the other samples tested also exhibited antifungal activity against the evaluated strains.

The Hexane Fraction (HF) and the SPF demonstrated low activity, with MIC and MFC values greater than 1000 μg/mL. However, the MPF, rich in myricitrin and gallic acid, as well as the LF, rich in ellagic acid, yielded the best results. Their MIC values ranged between 62.50 and 500 μg/mL (Table 4). Interestingly, when evaluated individually, the three polyphenols exhibited higher MIC and MFC values compared to their respective subfractions. Surprisingly, the combinations of these polyphenols did not demonstrate additive or synergistic effects.

The findings suggest that the presence of flavonoids significantly contributes to the antifungal properties of *E. uniflora* leaf extracts. In general, various antifungal mechanisms have been attributed to this important class of secondary metabolites. When tested against *Candida* spp. strains, these flavonoids inhibit processes such as cell wall formation, cell division, and the synthesis of RNA and proteins responsible for virulence factors.

Among the flavonoids individually evaluated in the literature, derivatives of myricitrin and kaempferol have demonstrated antifungal activity against strains of *C. albicans* [51] and *C. glabrata* [52,53]. Interestingly, the MPF contains derivatives of myricitrin and kaempferol, as well as quercetin and its derivatives (Table 3).

The other phenolic components present in LF, similar to EA, also yield considerable results. In general, phenols are associated with the antifungal action of herbal species due to their ability to induce membrane damage, resulting in an increase in cell permeability [54]. EA, which is present in LF, along with its glycosidic derivatives evaluated individually, has demonstrated antifungal activity against *C. albicans* and *C. auris* [55,56].

Species of the genus *Candida* spp. have significant clinical relevance due to their role as opportunistic pathogens in humans. Among the species evaluated, *C. albicans* is the most common, associated with frequent and recurrent vulvovaginal and oral infections, often exhibiting resistance to conventional antifungal drugs [9]. Although less prevalent, *C. glabrata* has emerged in healthcare settings, also demonstrating resistance to certain antifungal agents. At last, *C. auris*, a relatively recent addition to the list of pathogenic *Candida* species, has raised concern due to its multidrug-resistant nature, posing a serious threat to public health. In this context, the results of this study reinforce the promising potential of *E. uniflora* leaves, as already evidenced in the literature.

Therefore, the findings suggest that the concentration of the major polyphenols, myricitrin and ellagic acid, correlates with better antifungal responses. However, both compounds may act in conjunction with other components present in the phytocomplex, especially those with similar structures. Furthermore, the conducted in vitro tests offer crucial insights and suggest the potential for additional investigations into the specific mechanisms of antifungal action, including in vivo studies. These endeavors would yield more comprehensive data and validate the effectiveness of extracts, fractions, and isolates from *E. uniflora* leaves.

## 3. Material and Methods

### 3.1. Herbal Material, Extract, and Enriched Fraction

The leaves of *Eugenia uniflora* Linn were collected in the city of Paulista-PE and identified at the Agronomic Institute of Pernambuco (IPA) under registration number 93732 and registered with the National System for the Management of Genetic Heritage and Traditional Knowledge (Sisgen, Federal Republic of Brazil, Brazil) (number A449575). The material was dried in an air circulation oven (Luca-82-480^®^, Lucadema, São José do Rio Preto, São Paulo, Brazil) for 48 h at 40 °C. After drying, the leaves were ground in a Willye-type knife mill (TE-680^®^, Tecnal, Piracicaba, São Paulo, Brazil). The extractive solution was obtained by turboextraction at 10% (*w*/*v*), according to the published methodology [19] and spray-dried in a mini spray dryer (MSDi 1.0^®^, LabMaq, Ribeirão Preto, São Paulo, Brazil) under the following drying conditions: an inlet temperature of 150 °C, an airflow rate of 40 L/h, and a feed flow of 0.9 L/h to obtain the SDCE (18). The enriched fraction was obtained according to the LLE methodology described by Ramos, Bezerra [20]. The resulting fractions were concentrated under reduced pressure (RV10 Basic, IKA^®^), reconstituted with 50% (*w*/*v*) ethanol, and spray-dried in a mini spray dryer (MSDi 1.0, LabMaq^®^).

### 3.2. Screening Tests for Processing the Enriched Fraction

#### 3.2.1. Reversed-Phase Flash Chromatography

Around 0.2 g of the EAF was solubilized in 4 mL of distilled water and subjected to separation in an Isolera™ system (P/N 411829, Biotage^®^, Uppsala, Sweden) coupled to a variable-wave UV-Vis detector, set at 270 and 340 nm, and an automatic collector. The chromatographic separation was carried out using a Biotage^®^ SNAP-C_18_ column (cartridge size 25 g, average mass 30 g, and volume 33 mL) equipped with a pre-column of the same material. Two conditions were tested following the transposition of a methodology developed and validated on an analytical scale [17] and another with an extension of the analytical time. The sub-fractions were collected every 20 mL under a starting signal at a minimum detection of 5 mUA.

#### 3.2.2. Exclusion Chromatography

Initially, tests were carried out on an open glass column (h = 31 cm, Ø = 2 cm) packed with Sephadex^®^ LH-20 gel (MERCK^®^, Darmstadt, Germany) dispersed in an aqueous solution acidified with 2% (*v*/*v*) acetic acid. The final volume of the stationary phase was 50 mL, initially conditioned with 50% (*v*/*v*) methanol. Around 0.5 g of EAF was solubilized in 5 mL of the initial mobile phase and added to the top of the column. The mobile phase conditions evaluated in steps were: Test 1—40, 60, 80 and 100% (*v*/*v*) hydromethanolic solution; Test 2—40, 60, 80, and 100% (v/v) hydroethanolic solution; fractions of 5 mL were collected in Tests 1 and 2; Test 3—consisted of scaling up the technique to a glass column (h = 47 cm, Ø = 4 cm) with an stationary phase volume of 230 mL, previously conditioned with 40% (*v*/*v*) ethanol. Approximately 1 g of EAF solubilized in 5 mL of initial mobile phase was used and the elutions followed a gradient in steps: hydroethanolic solution 40, 60, 80 and 100% (*v*/*v*); Test 4—hydroethanolic solution 50, 80 and 100% (*v*/*v*). The sub-fractions from Tests 3 and 4 were collected at intervals of 10 mL of eluate. At the end of each test, the system was washed with acetone: water (7:3, *v*/*v*).

### 3.3. Chromatographic Strategies for Major Polyphenol Recovery

#### 3.3.1. EAF Processing by Subfractionation by Sephadex^®^ LH-20

Sephadex^®^ LH-20 (Sigma-Aldrich, San Luis, MO, USA) was suspended in 2% (*v*/*v*) acetic acid, and the resulting gel was packed into a glass column (h = 47 cm, Ø = 4 cm) to a volume of 230 mL, then the system was conditioned with 50% (*v*/*v*) ethanol. Chromatographic runs, called SPn, were carried out using approximately 1 g of EAF solubilized in 5 mL of 50% (*v*/*v*) ethanol for each run. The elutions followed the gradient of Test 5. Eluates of between 10 and 50 mL were collected. Similar subfractions, according to their evaluation during monitoring, were merged for processing in the following steps.

#### 3.3.2. EAF Processing by Subfractionation and Purification by Isolera™

The sub-fractions obtained in item 3.3.1 were subjected to separation by RP-FC using an Isolera™ system (Biotage—P/N 411829) coupled to a variable-wave UV-Vis detector and automatic collector. The chromatographic separations were carried out using a Biotage^®^ SNAP-C_18_ column (cartridge size of 10 or 25 g, average mass of 12 or 30 g, and volume of 15 or 33 mL, respectively), with a pre-column suitable for each cartridge size and made of the same material. The eluent consisted of distilled water and methanol, acidified with 0.1% (*v*/*v*) acetic acid, as mobile phases A and B, respectively. Chromatographic conditions such as column size, gradient, and flow were modified and optimized for each constituent during the processing of the subfractions, as were the set wavelength, starting detection limits, and collection volume (Table 5).

Exceptionally, an additional sample preparation procedure was adopted for the recovery of the flavonoid myricitrin. Approximately 50 mg of MPF was solubilized in 5 mL of the initial mobile phase B 10% (*v*/*v*) using an ultrasonic bath (Cristófoli^®^, Campo Mourão, Paraná, Brazil). The solution was then subjected to 2000 rpm for 5 min in a centrifuge (EEQ9004A-2, 9TEC^®^, Curitiba, Paraná, Brazil), and the resulting supernatant was subsequently subjected to RP-FC. The precipitate was solubilized under the described conditions and centrifuged again; this procedure was repeated until no more precipitate was observed.

#### 3.3.3. Yields

All the subfractions obtained were placed at 40 ± 2 °C to evaporate the solvent. After drying, each subfraction was resuspended in methanol, and the solution was transferred to a pre-weighed vial. The vials were then stored uncapped at room temperature (30 °C) until the solvent had completely evaporated. The vials containing the dried subfractions were weighed, and the yields were calculated as a percentage. This calculation took into account the weight of each subfraction, the weight of the sample that was used, named yield (Y%), and the total weight of all the subfractions, named relative yield (RY%), for the tests in SEC. For the RP-FC tests, the weights of the samples used were compared with the weight of the sub-fraction collected.

### 3.4. Monitoring the Sub-Fractionation and Isolation Process

#### 3.4.1. Thin Layer Chromatography (TLC)

The samples obtained during the process were resuspended in methanol, and small volumes were applied to 60-F_254_ silica gel plates with 10—12 μm particles (Merck KGaA^®^, Darmstadt, Germany) using a glass capillary. The chromatograms were developed in a vertical glass chamber with a double trough (20 cm × 10 cm, Camag^®^, Muttenz, Switzerland) after being saturated for 30 min with the 90:5:5 mobile phase (ethyl acetate: formic acid: water, *v*/*v*/*v*). Following this process, the TLC plates were derivatized with the reagent aluminum chloride (AlCl_3_) at 5% (*w*/*v*) and evaluated under UV light at 365 nm for flavonoids. They were also derivatized with ferric chloride (FeCl_3_) at 5% (*w*/*v*) for hydrolysable tannins and evaluated under visible light. Image acquisition and UV observations were carried out using the MultiDoc—It Imaging System^®^ (Model 125) with UVP^®^ software and a Canon^®^-coupled camera (Rebel T3, EOS 1100 D, Canon, Tokyo, Japan).

#### 3.4.2. High Performance Liquid Chromatography (HPLC-DAD)

The samples were analyzed by HPLC using an Ultimate 3000 system (Thermo Scientific^®^, Waltham, MA, USA), equipped with a diode array detector (DAD; Thermo Fisher Scientific^®^, Waltham, MA, USA). Chromeleon software (Dionex^®^, version 6.0) was utilized for data acquisition and processing. Chromatographic separation was performed using a C_18_ column (250 mm × 4.6 mm i.d., 5 μm; Supelco^®^, Sigma-Aldrich, San Luis, MO, USA ), which was equipped with a pre-column of the same material (4 mm × 3.9 μm, Phenomenex^®^, Torrance, CA, USA). The analysis conditions that were described and validated previously were employed [19].

### 3.5. Characterization LC-ESI-HRMS/MS

EAF, SPF, MPF, and LF were analyzed by Ultra-Performance Liquid Chromatography (UPLC) using a Prominence UFLC system (Shimadzu^®^, Quioto, Japan), equipped with a diode array detector (SPD-M20A, Shimadzu^®^), and coupled to a MicrO-TOF-QII™ mass detector (Bruker^®^, Billerica, MA, USA). Chromatographic separation was performed using a C_18_ column (100 mm × 4.6 mm i.d., 3.5 μm, WaterS Symmetry^®^, Milford, MA, USA) at an oven temperature of 25 °C. The mobile phase comprised solvent A (purified water, Milli-Q^®^, Merck Group, Darmstadt, Germany) and solvent B (methanol, LC-MS grade), both acidified with 0.1% formic acid (HPLC grade), and flowed at a rate of 0.5 mL/min^−1^, following the gradient: 0–2 min (10% B), 2–40 min (40–75% B), 40–48 min (75% B), 48–48.5 min (75–10% B), and 48.5–50 min (10% B). The MS parameters used were: electrospray ionization (ESI) at negative ion polarity; capillary voltage 2.3 kV and end plate offset −500 V; nebulizer pressure 2.0 Bar; dry gas flow 10.0 L.min^−1^ and dry heater 200 °C. The radio frequency in the collision cell was maintained at 200 Vpp, and the MS/MS collision energy was 35 eV. The isolates were evaluated by direct injection into a MicrO-TOF-QII™ mass spectrometer (Bruker^®^, Billerica, MA, USA). The equipment utilized electrospray ionization (ESI) under the following operating conditions: negative ion polarity; capillary voltage of 2.3 kV and end plate offset of −500 V; nebulizer pressure of 1.0 Bar; dry gas flow of 4.0 L.min^−1^ and dry heater at 180 °C. The radio frequency in the collision cell was maintained at 250 Vpp. The samples were introduced with the help of an automatic syringe pump, using approximately 10 μg/L samples at a constant flow of 180 μL/h of MS-grade methanol. The spectra were obtained in the 100–1000 *m*/*z* scanning range, processed in the Bruker DataAnalysis^®^ 4.0 software, and expressed as *m*/*z*.

### 3.6. Minimum Inhibitory Concentration (MIC) and Minimum Fungicidal Concentration (MFC)

The antifungal evaluation of SDCE, fractions, subfractions, and isolates was conducted using standard strains of *Candida albicans* (90028), *Candida glabrata* (9001), and *Candida auris* (CDC B11903) from the American Type Culture Collection (ATCC). The yeasts were manipulated under sterile experimental conditions and grown on Sabouraud agar for 48 h at 37 °C. The fungal suspension was obtained on the 0.5 McFarland scale by adjusting the turbidity to 530 nm on a spectrophotometer (Micronal^®^, AJX1900, Tecnal, Piracicaba, São Paulo, Brazil). After the inoculums were calibrated, two dilutions were made: one of 1:50 in sterile saline solution and a further dilution of 1:20 in culture medium, yielding a final concentration of 10^3^ CFU/mL. The tests were carried out in a 96-well microplate, using 100 μL of the culture medium in all the wells and 100 μL of the sample, each sample in the well of its corresponding row. Serial dilutions were made in the following wells, discarding 100 μL from the well of column 10, obtaining concentrations of 1000 to 1.95 μg/mL. A total of 100 μL of the inoculum was added to the wells of columns 1 to 11. In column 11, the presence of inoculum and absence of a sample served as a positive control, facilitating the observation of inoculum viability. In column 12, 200 μL of culture medium without sample or inoculum was added, constituting the negative control. The plates were incubated for 48 h at a temperature of 30–37 °C, after which they were examined for the presence or absence of growth. The MIC was determined by the lowest concentration at which each sample could inhibit fungal growth, in comparison to the positive control. To determine the MFC, an aliquot of 5 μL was taken from each well of the MIC test and transferred to a Petri dish containing Sabouraud agar, then incubated for 48 h at 37 °C. The MFC was evaluated at the lowest concentration at which each sample could prevent visible fungal growth (CLSI).

## 4. Conclusions

The fractions and crude extract of *E. uniflora* leaves demonstrated anti-*Candida* activity against *C. albicans*, *C. glabrata*, and *C. auris*, consistent with findings in the literature. The partitioning and purification strategy facilitated the extraction of primary phyto-chemicals, revealing that certain compounds, such as hydrolysable tannin ellagic acid and the flavonoid myricitrin, were associated with the biological response of these ex-tracts. This discovery carries significant implications, offering valuable insights and guiding future research endeavors aimed at developing and standardizing new antifungal products derived from plant species rich in these classes of metabolites. The fractions enriched with myricitrin and ellagic acid exhibited yields of 10.36% and 1.19%, respectively, indicating the feasibility of obtaining sub-fractions and purified compounds from *E. uniflora* leaves for research purposes, although not immediately scalable for incorporation as active ingredients in new products. The MICs ranged from 62.5 to 125.0 for the fractions enriched with myricitrin and ellagic acid, respectively, underscoring the potential and influence of these constituents on the antifungal activity of the extracts. Therefore, further studies are warranted to elucidate the mechanisms of action involved and explore the potential contributions of other phytochemicals described in the matrix.

## Figures and Tables

**Table 1:** Yield of fractions obtained from SDCE by LLE.

	Sample (g)			Yields %		
	SDCE	AqF	EAF	AqF	EAF/AqF	EAF/SDCE
1	360	177	7.87	49.16	4.44	2.19
2	270	110	6.14	40.74	5.58	2.27
3	180	78	4.27	43.33	5.47	2.37
Mean ± SD (RSD%)				43.33 ± 4.31 (9.95)	5.47 ± 0.62 (11.49)	2.27 ± 0.09 (3.97)

SDCE—spray-dried crude extract; AqF—aqueous fraction; EAF—ethyl acetate fraction; SD—standard deviation; RSD%—relative standard deviation.

**Table 2:** Yields of subfractions collected in SP1 from SEC.

SP1				
nFr	Eluate (mL)	Weight (mg)	Y%	RY%
1	50	32.90	3.21	3.26
2	30	144.10	14.05	14.30
3	20	63.70	6.21	6.32
4	20	30.00	2.92	2.98
5	20	23.50	2.29	2.33
6	20	51.70	5.04 #	5.13
7	20	42.90	4.18 #	4.26
8	20	79.20	7.72 #	7.86
9	20	47.70	4.65 #	4.73
10	25	20.30	1.98 #	2.01
11	20	19.80	1.93	1.96
12	20	14.10	1.37	1.40
13	20	11.60	1.13	1.15
14	20	12.00	1.17	1.19
15	25	14.60	1.42	1.45
16	25	15.40	1.50	1.53
17	25	17.10	1.67	1.70
18	50	25.90	2.53	2.57
19	55	53.40	5.21	5.30
20	65	19.00	1.85 *	1.89
21	50	8.70	0.85 *	0.86
22	100	36.80	3.59	3.65

# yield% of the subfractions rich in gallic acid and myricitrin (MPF); * yield of the subfractions with ellagic acid (LF). nFr—subfraction number; EAF—ethyl acetate fraction; Y%—yield in percentage considering the amount of EAF; RY%—yield in percentage considering the sum weight of the subfractions.

**Table 3:** Characterization of the compounds identified by HPLC-DAD-ESI-HRMS/MS in *Eugenia uniflora*.

Peak No.	*t*_R_ (min.)	*m*/*z* [M − H]^−^	Molecular Formula	Error (ppm)	MS/MS (Relative Abundance%)	Tentative Assignment	Fraction	Reference
1	2.2	191.0562	C_7_H_12_O_6_	−0.6	173.0954 (23.81); 165.0385 (19.05); 127.0367 (45.24)	Quinic acid	SPF/EAF	[15,44,45,46]
2	4.6	191.0197	C_6_H_8_O_7_	0.2	173.0078 (73.33); 111.0086 (60.00)	Citric acid	SPF/MPF/EAF	[47]
3	6.3	169.0149	C_7_H_6_O_5_	−3.8	125.0244 (100.00)	Galic acid*	SPF/MPF/EAF	[15,44,46]
4	15.2	483.0788	C_20_H_20_O_14_	−1.7	313.0595 (17.37); 271.0433 (0.47); 211.0230 (2.11); 169.0149 (77.43); 125.0264 (0.43)	Digalloyl-hexoside	SPF	[15,44,45,46]
5	15.2	353.0889	C_16_H_18_O_9_	−3.2	191.0543 (100.00)	5-*O*-Caffeoylquinic acid	SPF/EAF	[44,46]
6	19.2	337.0928	C_16_H_18_O_8_	0.1	191.0529 (100.00); 163.0380 (14.34); 119.0491(8.93)	5-*O*-Coumaroylquinic acid	SPF/EAF	[15,46]
7	24.2	653.2130	C_30_H_38_O_16_	2.4	501.1996 (44.29); 483.1829 (19.36); 313.0577(14.16); 271.0507 (3.87); 211.0194 (7.37); 193.0133 (4.53); 169.0153 (54.58); 125.0216 (6.99)	Digalloyl-hexoside derivative	SPF/EAF	[48]
8	25.1	479.0848	C_21_H_20_O_13_	−3.5	317.0245 (24.83); 316.0230 (100.00); 287.0196 (7.65); 271.0269 (13.79); 178.9984 (2.67); 151.0062 (3.39)	Myricetin-*O*-hexoside	MPF	[15,44,45,46]
9	25.2	653.2084	C_30_H_38_O_16_	0.5	501.2041 (43.42); 483.1850 (30.92); 313.0568 (23.68); 241.0370 (11.84); 169.0177 (100.00)	Digalloyl-hexoside derivative	MPF	[48]
10	25.4	501.1983	C_23_H_34_O_12_	−1.1	451.3264 (25.87); 313.0593 (15.85); 271.0444 (12.63); 211.0243 (9.84); 169.0138 (59.41); 125.0236 (10.19)	Digalloyl-hexoside derivative	SPF	[48]
11	25.4	539.2142	C_23_H_36_O_12_	−1.4	501.1896 (9.52); 313.0560 (6.54); 271.0440 (4.93); 169.0147 (46.44); 125.0235 (15.25)	Digalloyl-hexoside derivative	SPF	[48]
12	25.5	449.0731	C_20_H_18_O_12_	−1.2	317.0267 (25.26); 316.0229 (100.00); 287.0193 (13.96); 271.0245 (21.05); 178.9980 (4.98); 151.0067 (2.28)	Myricetin-*O*-pentoside	MPF	[15,44,45,46]
13	26.6	449.0728	C_20_H_18_O_12_	−0.6	317.0251 (21.11); 316.0225 (100.00); 287.0193 (14.18); 271.0242 (27.36); 178.9992 (2.01); 151.0019 (3.45)	Myricetin-*O*-pentoside	MPF	[15,44,45,46]
14	27.1	463.0900	C_21_H_20_O_12_	−3.9	317.0265 (20.75); 316.0223 (100.00); 287.00201 (8.96); 271.0243 (19.39); 178.9995 (3.86); 151.0046 (3.38)	Myricetin-*O*-rhamnoside * (Myricitrin)	MPF	[15,44,46]
15	28.0	463.0875	C_21_H_20_O_12_	1.6	301.0322 (33.21); 300.0263 (100.00); 271.0232 (32.62); 255.0293 (14.11); 179.0010 (4.51); 151.0071 (4.72)	Quercetin-*O*-hexoside	MPF	[15,45]
16	28.3	463.0876	C_21_H_20_O_12_	1.3	301.0319 (35.81); 300.0264 (100.00); 271.0228 (37.49); 255.0310 (25.03); 178.9933 (10.66); 151.0052 (8.38)	Quercetin-*O*-hexoside	MPF	[15,45]
17	28.9	433.0773	C_20_H_18_O_11_	0.8	301.0308 (28.23); 300.0270 (100.00); 271.0214 (40.46); 255.0279 (27.53); 178.9937 (3.17); 151.0023 (4.13)	Quercetin-*O*-pentoside	MPF	[15,45]
18	30.1	433.0771	C_20_H_18_O_11_	1.2	301.0337 (33.29); 300.0274 (100.00); 271.0261 (33.82); 255.0314 (23.84); 179.0014 (3.36); 151.0080 (8.93)	Quercetin-*O*-pentoside	MPF	[15,45]
20	30.5	447.0941	C_21_H_20_O_11_	−1.8	301.0324 (60.54); 300.0266 (100.00); 271.0244 (42.29); 255.0294 (18.59); 178.9983 (6.63); 151.0042 (11.36)	Quercetin-*O*-deoxyhexoside	MPF	[15,45]
21	33.2	431.0992	C_21_H_20_O_10_	−1.8	285.0404 (75.39; 284.0299 (100.00); 255.0314 (86.57); 227.0335 (81.93)	Kaempferol-*O*-deoxyhexoside	MPF	[15,45]
22	33.3	471.1305	C_24_H_24_O_10_	−1.7	285.0368 (10.07); 284.0314 (14.76); 255.0298 (1.50); 227.0376 (2.45)	Kaempferol- derivative	MPF	-

* Compared with standard.

**Table 4:** MIC and MFC results of the extract, fractions, subfractions, and isolates of *E. uniflora* leaves.

Minimum Inhibitory Concentration (MIC) and Minimum Fungicidal Concentration (MFC) (μg/mL)						
	*Candida albicans*		*Candida glabrata*		*Candida auris*	
Samples	MIC	MFC	MIC	MFC	MIC	MFC
SDCE	250 *	500	125 *	≥1000	31.2 *	1000
Fractions						
* HF*	1000	1000	1000	1000	62.5 *	1000
* EAF*	250*	500	125 *	1000	31.2 *	1000
* rFaq*	1000	1000	250	1000	125	1000
* SPF*	1000	≥1000	1000	≥1000	500	1000
* MPF*	125 *	125	62.5 *	250	500	1000
* MyR I*	250 *	500	250 *	500	500	≥1000
* GA I*	500	500	250 *	500	500	≥1000
* LF*	125 *	125	125 *	250	125 *	1000
* EA I*	250	250	62.5 *	250	125 *	1000
Phytochemicals						
* GA*	500	≥1000	250 *	500	500	500
* MyR*	500	1000	250 *	250 *	500	1000
* EA*	500	1000	125 *	250 *	1000	1000
Synergic samples						
* GA + MyR*	500	500	125 *	500	1000	1000
* MyR + EA*	500	1000	125 *	500	500	≥1000
* EA + GA*	1000	≥1000	1000	≥1000	1000	≥1000
* GA + EA + MyR*	1000	≥1000	500	≥1000	1000	≥1000

MIC—Minimum Inhibitory Concentration; MFC—Minimum Fungicidal Concentration; CE—crude extract; HF—hexane fraction; EAF—ethyl acetate fraction; rFaq—residual aqueous fraction; SPF—fraction of cinnamic derivatives; MPF—flavonoid fraction; MyR I—myricitrin-rich fraction; LF—last fractions collected on Sephadex^®^ LH-20; EA I—ellagic acid-rich fraction; GA—gallic acid isolate; MyR—myricitrin isolate; EA—ellagic acid isolate. * MIC with considerably significant results.

**Table 5:** Chromatographic conditions in RP-FC for the recovery of majority polyphenols.

		Recovery of EA				Recovery of GA				Recovery of MyR					
Processed Samples		LF		EA I		MPF		GA I		MPF (SNT1)		MyR I (SNT2)		MyR II (SNT3)	
Chromatographic conditions in RP-FC	Gradient	B%	min	B%	min	B%	min	B%	min	B%	min	B%	min	B%	min
		10	2	10	3	10	4	10	4	10	7	30	1	50	3
		20	3	30	5	10–40	10	100	2	10–100	20	30–70	6	50–56	2
		30	3	30–100	1	40–100	6	10	2	100–10	1	100	3	56–100	2
		30–100	1	100	5	100–10	1	-	-	10	3	30	1	100	2
		100	3	-	-	10	4	-	-					50	2
	Flow (mL/min)	12		12		15		20		20		20		20 e 15	
	Column (g)	12		12		12		30		30		30		30 e 12	
	λ (nm)	254 and 280				254 and 270				270 and 350					
	ST (mAU)	10		10		20		5		10		5		5	
	max. vol (mL)	10		10		15		10		15		10		10	

B%—proportion of mobile phase B; LF—last fractions; EA I—ellagic acid subfraction; EA—purified ellagic acid after processing EA I in isolera; GA I—gallic acid subfraction after processing MPF in isolera; GA—purified gallic acid after processing GA I in isolera; MPF—main polyphenols subfraction; MyR I—myricitrin-rich subfraction; MyR II—subfraction obtained after processing MyR I; SNT—supernatant; λ—wavelength; ST—starting detection; max vol—maximum collection volume.

## Data Availability

Data are contained within the article.

## Supplementary Materials

The following supporting information can be downloaded at: https://www.mdpi.com/article/10.3390/molecules29122761/s1, Figure S1. Chromatographic profile of spray-dried crude extract (SDCE) from E. uniflora leaves at 270 nm (A) and chomatograms of the Ethyl Acetate Fraction (EAF) and the crude extract (SDCE) compared at 270 nm. Figure S2. Chromatogram of test 1 of the RP-FC screening (A) and TLCs of the collected sub-fractions derivatized with 5% (*w*/*v*) AlCl3 (B) and FeCl3 (C). Figure S3. Absorption spectrum of the peak with retention time of 23,91 min from Test 1 in SEC screening corresponding to EA. Figure S4. TLC of the subfractions collected in test 2 of the SEC screening derivatized with 5% (*w*/*v*) AlCl3. Figure S5. Chromatograms of subfraction 16 at 270 nm (A), along with the respective UV spectra of GA and peak 2 (A); chromatograms of subfraction 20 at 340 nm, and the respective UV spectra of MyR and peaks 1 and 3 (B); chromatograms of subfractions 40, 44, and 48 at 254 nm, and the respective UV spectra of EA and peaks 1 and 2. Test 2 samples in SEC. Figure S6. Monitoring TLCs of the subfractions collected from test 3 by SEC derivatized with 5% (*w*/*v*) AlCl3. Figure S7. Monitoring TLCs of the subfractions collected from test 4 by SEC derivatized with 5% (*w*/*v*) AlCl3. Figure S8. Monitoring TLCs of the subfractions collected in SEC fractionations derivatized with 5% (*w*/*v*) AlCl3. Table S1. Yields of sub-fractions collected in Test 4 from SEC. Table S2. Yields of the sub-fractions collected in the process of obtaining EA II. Table S3. Yields of the subfractions collected in the process of obtaining GAF from FLAVF. Figure S9. TLC of precipitate (P) and edge (B) samples from MyR I and II suffractions derivatized with 5% (*w*/*v*) AlCl3. Table S4. Yields of subfractions collected when processing MyR I and II subfractions. Figure S10. Spectrometric profile of myricitrin (A), gallic acid (GA) and ellagic acid (C) isolates from E. uniflora leaves evaluated by direct injection in ESI-HRMS/MS.
