# Supplementary material for: Influence of Major Polyphenols on the Anti-Candida Activity of Eugenia uniflora Leaves: Isolation, LC-ESI-HRMS/MS Characterization and In Vitro Evaluation
Source: Molecules. 2024 Jun 10;29(12):2761. doi: 10.3390/molecules29122761 (PMC11206001; doi:10.3390/molecules29122761)
Supplement: Supplementary file 1 [file molecules-29-02761-s001.zip › molecules-3017984-supplementary.pdf]

Supplementary Material

Influence of major polyphenols on the of anti-*Candida* activity of *Eugenia uniflora* leaves: isolation, characterization LC-ESI-HRMS/MS and *in vitro* evaluation

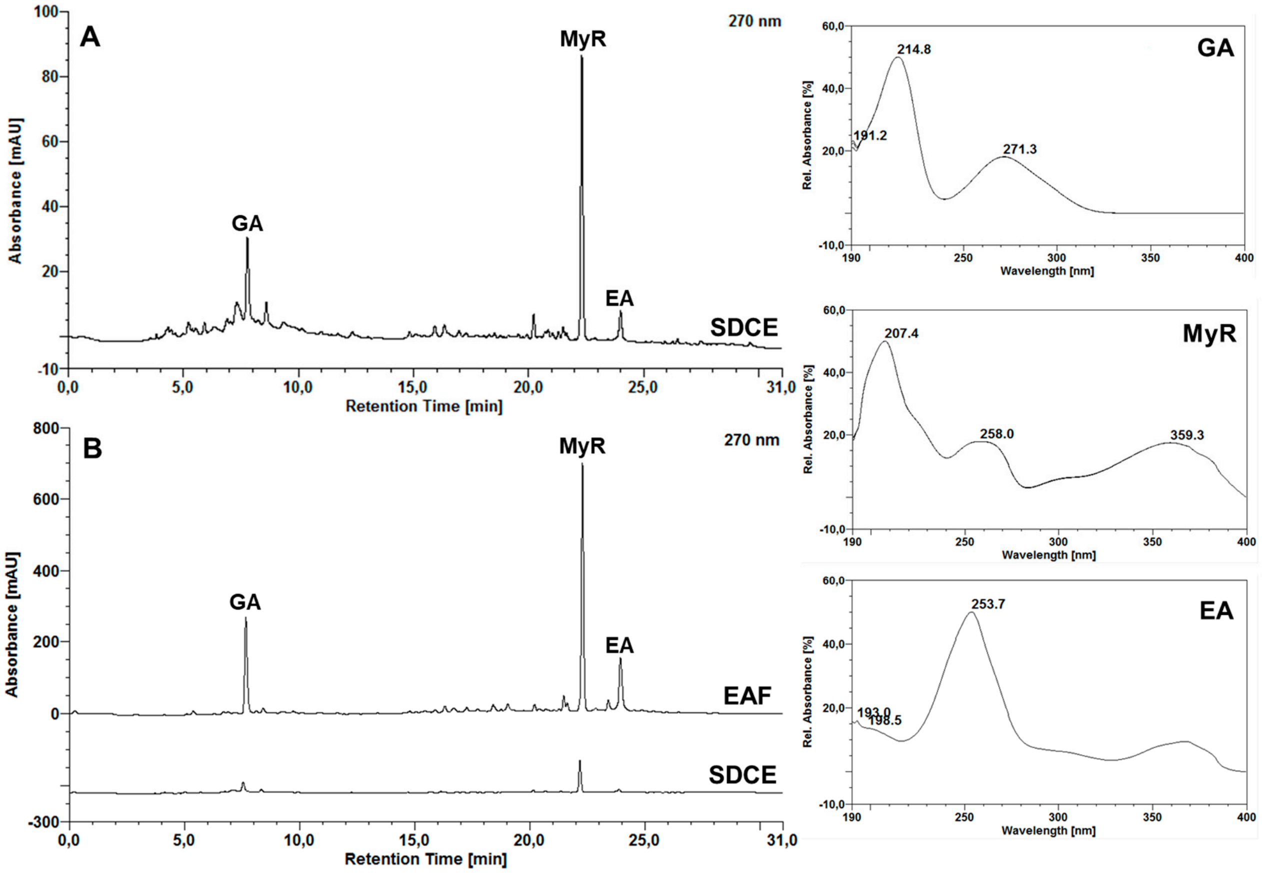

Figure S1. Chromatographic profile of spray-dried crude extract (SDCE) from *E. uniflora* leaves at 270 nm (**A**) and chomatograms of the Ethyl Acetate Fraction (EAF) and the crude extract (SDCE) compared at 270 nm (**B**).

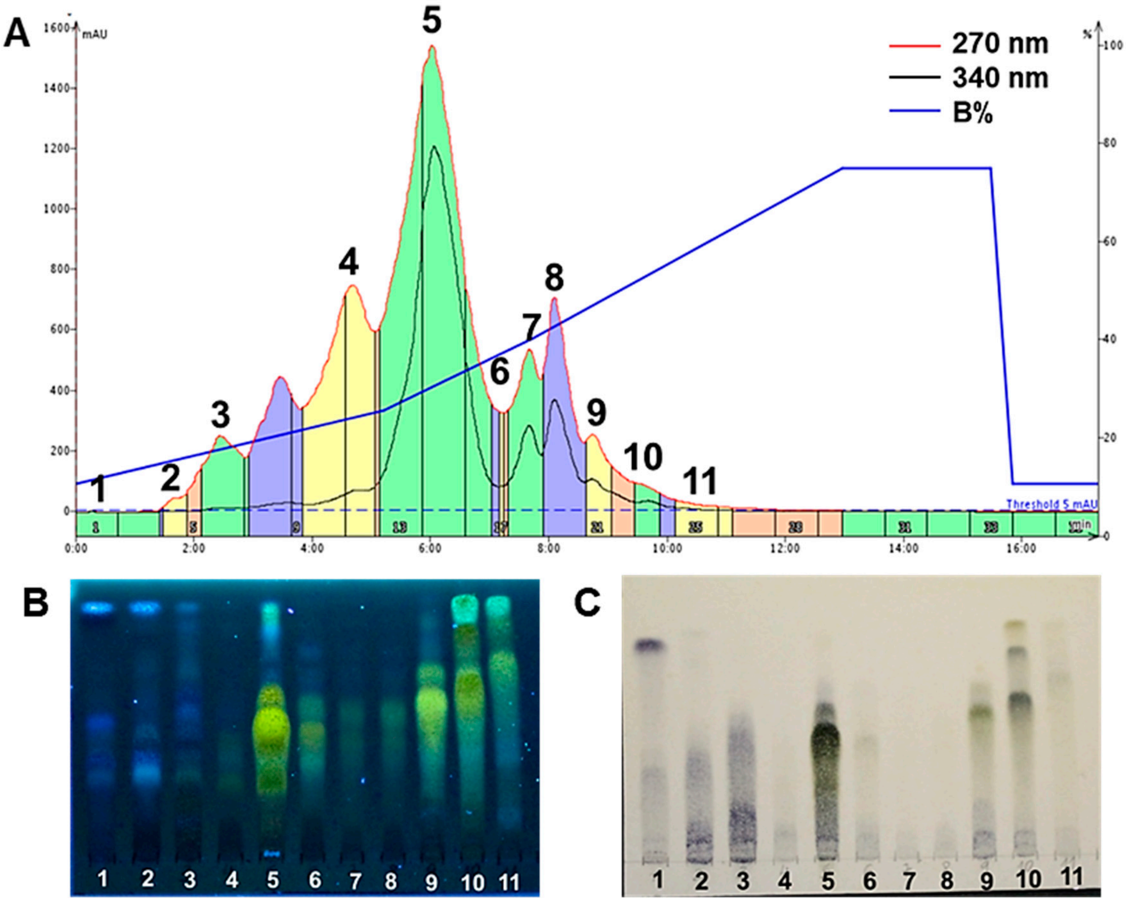

Figure. S2. Chromatogram of test 1 of the RP-FC screening (**A**) and TLCs of the collected sub-fractions derivatized with 5% (w/v) $AlCl_3$ (**B**) and $FeCl_3$ (**C**).

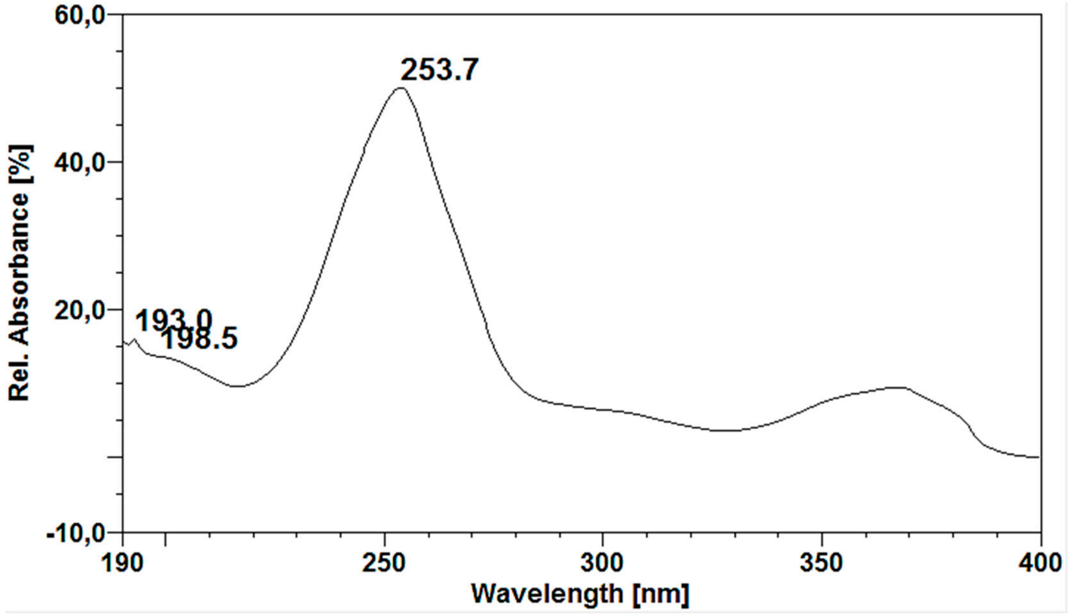

Figure. S3. Absorption spectrum of the peak with retention time of 23,91 min from Test 1 in SEC screening corresponding to EA.

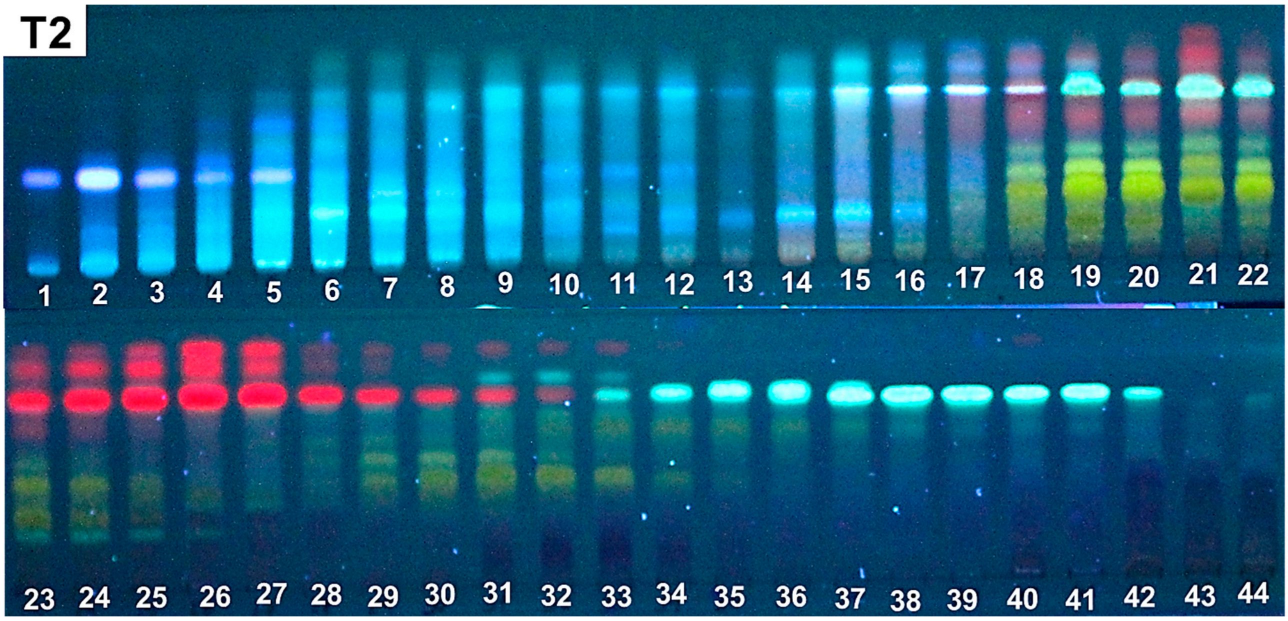

Figure. S4. TLC of the subfractions collected in test 2 of the SEC screening derivatized with 5% (w/v) $AlCl_3$.

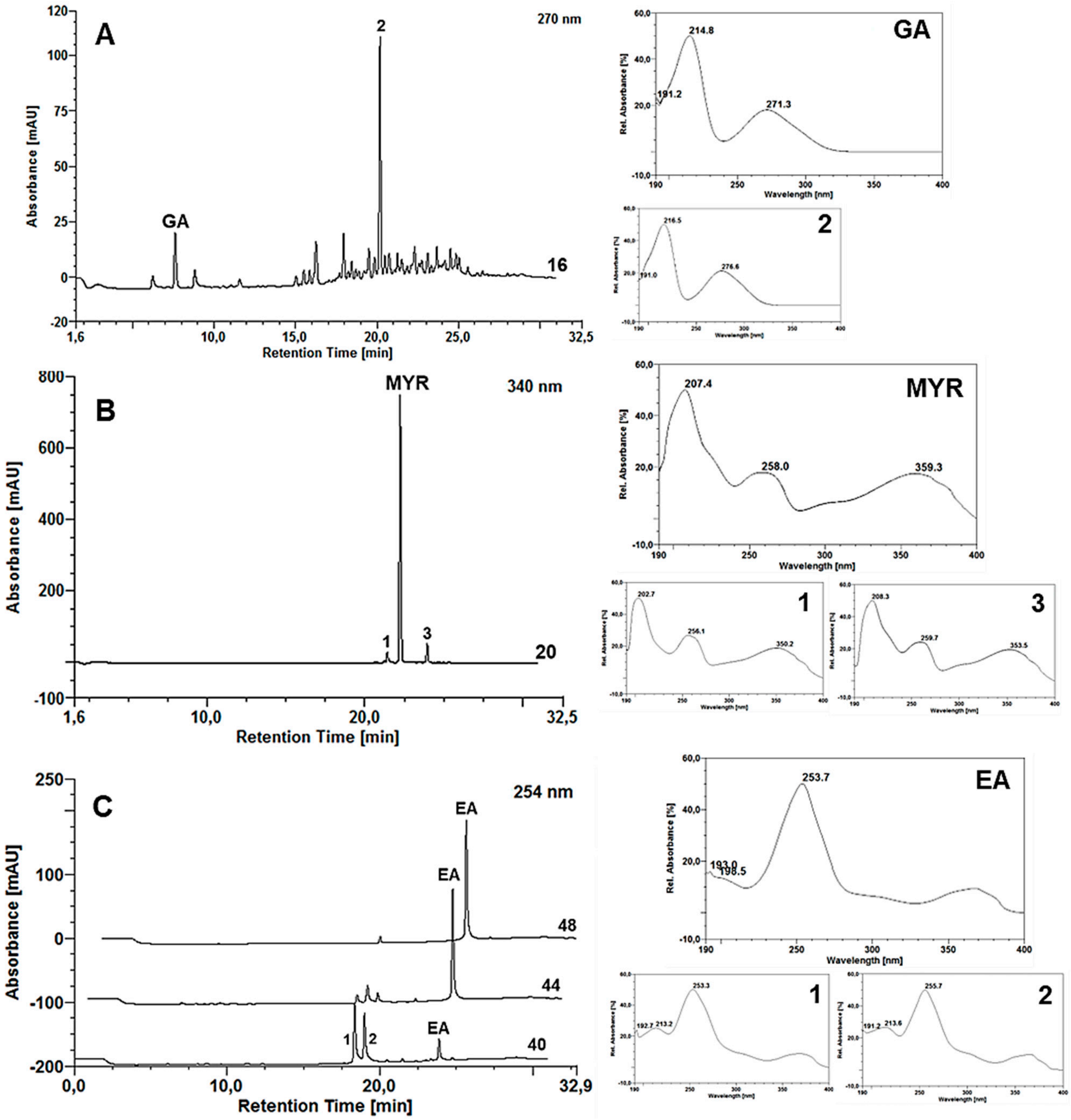

Figure. S5. Chromatograms of subfraction 16 at 270 nm (**A**), along with the respective UV spectra of GA and peak 2 (**A**); chromatograms of subfraction 20 at 340 nm, and the respective UV spectra of MyR and peaks 1 and 3 (**B**); chromatograms of subfractions 40, 44, and 48 at 254 nm (**C**), and the respective UV spectra of EA and peaks 1 and 2. Test 2 samples in SEC (**C**).

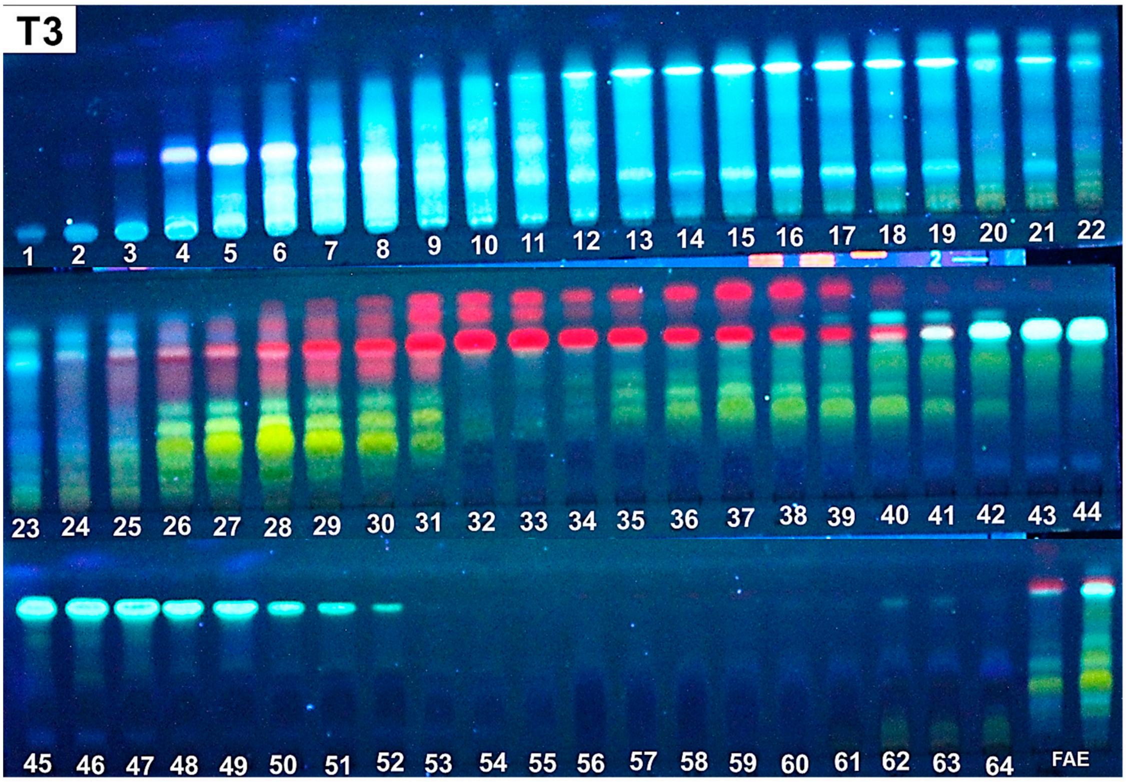

Figure. S6. Monitoring TLCs of the subfractions collected from test 3 by SEC derivatized with 5% (w/v) $AlCl_3$.

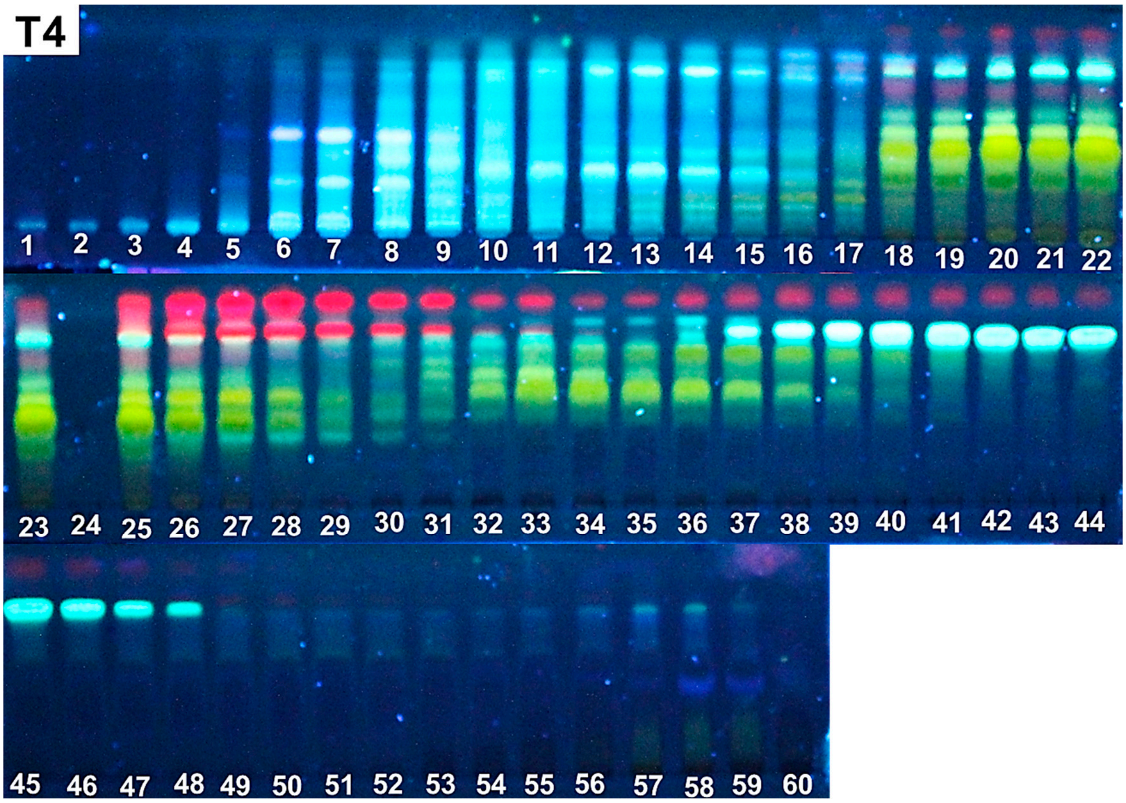

Figure. S7. Monitoring TLCs of the subfractions collected from test 4 by SEC derivatized with 5% (w/v) $AlCl_3$.

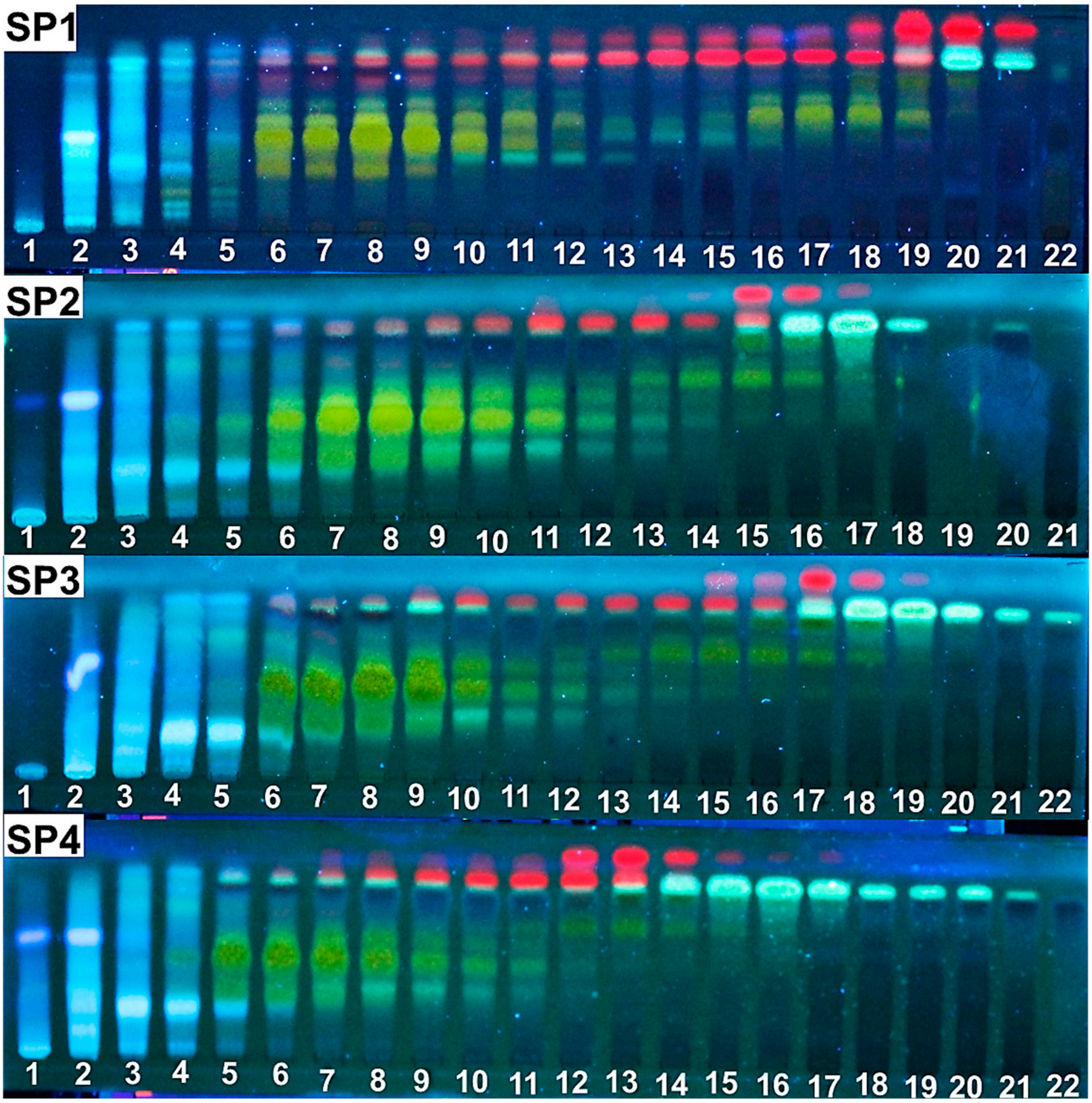

Figure. S8. Monitoring TLCs of the subfractions collected in SEC fractionations derivatized with 5% (w/v) $AlCl_3$.

Table. S1. Yields of sub-fractions collected in Test 4 from SEC.

T4											
**nFr**	**weight(mg)**	**Y%**	**RY%**	**nFr**	**weight(mg)**	**Y%**	**YY%**	**nFr**	**weight(mg)**	**Y%**	**RY%**
**1**	0.69	0.03	0.03	**21**	75.20	3.74	3.76	**41**	10.10	0.50	0.50
**2**	0.70	0.03	0.03	**22**	67.70	3.36	3.38	**42**	6.90	0.34	0.34
**3**	1.70	0.08	0.08	**23**	66.40	3.30	3.32	**43**	5.10	0.25	0.25
**4**	7.10	0.35	0.35	**24**	-	-	-	**44**	7.30	0.36	0.36
**5**	24.80	1.23	1.24	**25**	70.40	3.50	3.52	**45**	5.20	0.26	0.26
**6**	55.60	2.76	2.78	**26**	36.50	1.81	1.82	**46**	3.50	0.17	0.17
**7**	56.60	2.81	2.83	**27**	21.90	1.09	1.09	**47**	2.90	0.14	0.14
**8**	92.20	4.58	4.61	**28**	17.10	0.85	0.85	**48**	3.50	0.17	0.17
**9**	65.30	3.25	3.26	**29**	15.80	0.79	0.79	**49**	4.20	0.21	0.21
**10**	57.30	2.85	2.86	**30**	16.30	0.81	0.81	**50**	5.00	0.25	0.25
**11**	39.70	1.97	1.98	**31**	16.40	0.82	0.82	**51**	6.10	0.30	0.30
**12**	39.20	1.95	1.96	**32**	17.60	0.87	0.88	**52**	4.40	0.22	0.22

**13**	33.00	1.64	1.65	**33**	19.20	0.95	0.96	**53**	7.30	0.36	0.36
**14**	25.50	1.27	1.27	**34**	19.00	0.94	0.95	**54**	9.60	0.48	0.48
**15**	23.10	1.15	1.15	**35**	18.30	0.91	0.91	**55**	15.30	0.76	0.76
**16**	21.10	1.05	1.05	**36**	16.00	0.80	0.80	**56**	19.70	0.98	0.98
**17**	17.40	0.86	0.87	**37**	13.80	0.69	0.69	**57**	156.10	7.76	7.80
**18**	40.60	2.02	2.03	**38**	11.30	0.56	0.56	**58**	287.00	14.26	14.34
**19**	59.80	2.97	2.99	**39**	8.70	0.43	0.43	**59**	128.50	6.39	6.42
**20**	81.60	4.06	4.08	**40**	9.20	0.46	0.46	**60**	33.90	1.68	1.69

#yield% of the subfractions rich in gallic acid and myricitrin (MPF); *yield of the subfractions with ellagic acid (LF).
nFr - subfraction number; EAF - ethyl acetate fraction; Y% - yield in percentage considering the amount of EAF; RY% - yield in percentage considering the sum weight of the subfractions.

Table. S2. Yields of the sub-fractions collected in the process of obtaining EA II.

	**Recovery of EA I**						**Recovery of EA II**		
**Qt. LF (mg)**	72.90		109.10		101.20		**Qt. EA I (mg)**	102.10	
Rep.	1		2		3				
nFr	Yield (mg)	Y%	Yield (mg)	Y%	Yield (mg)	Y%	**nFr**	Yield (mg)	Y%
**1**	0.23	0.32	0.51	0.47	0.36	0.36	**1**	0.23	0.23
**2**	1.21	1.66	3.89	3.57	2.78	2.75	**2**	0.11	0.11
**3**	26.37	36.17	38.31	35.11	26.49	26.18	**3 (EA II)**	12.1	11.85
**4 (EA I)**	1.55	2.13	3.84	3.52	1.24	1.23	**4**	0.07	0.07
**5**	0.35	0.48	0.93	0.85	0.14	0.14			

EAF - ellagic acid fraction; LF - last fractions; nFr - subfraction number; Y% - yield in percentage; Rep -repetition; Qt. - quantity.

Table. S3. Yields of the subfractions collected in the process of obtaining GAF from FLAVF.

	**Recovery of GA I**					
**Qt. MPF (mg)**	200,00		200,00		150,00	
Rep.	1		2		3	
nFr	Yield (mg)	Y%	Yield (mg)	Y%	Yield (mg)	Y%
**1 (GA I)**	71.50	35.75	34.00	17.00	43.60	29.10
**2**	1.30	0.65	2.80	1.40	6.30	4.20
**3**	1.20	0.60	42.30*	21.15	20.00*	13.33
**4**	7.10	3.55	6.30	3.15	16.00#	10.67
**5**	38.80*	19.40	27.80#	13.90	3.80	2.53
**6**	12.90#	6.45	13.10	6.55		
**7**	8.00	4.00	7.30	3.65		
**8**	1.60	0.80				

*second major peak of the chromatogram; #third major peak of the chromatogram.
MPF – mais polyphenols subfraction; GA I - gallic acid fraction; nFr - subfraction number; Y% - yield in percentage; Rep - repetition; Qt. - quantity.

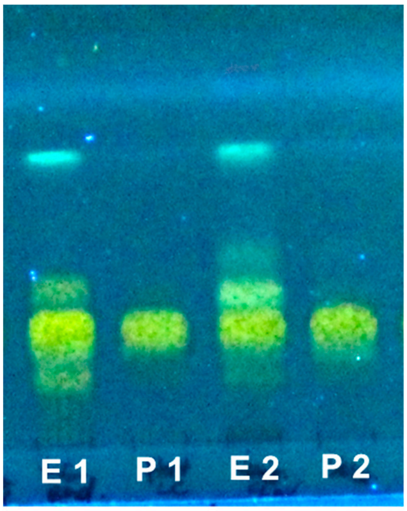

Figure. S9. TLC of precipitate (P) and edge (B) samples from MyR I and II suffractions derivatized with 5% (w/v) $AlCl_3$.

Table. S4. Yields of subfractions collected when processing MyR I and II subfractions.

**Recovery of MyR subfractions**									
**Qt. (mg)**	MPF	SNT1		Precip.1	SNT2		Precip.2	SNT3	
	52.3	35.94		13.36	6.37		9.99	9.71	
	1			2			3		
	nFr	Yield (mg)	Y%	nFr	Yield (mg)	Y%	nFr	Yield (mg)	Y%
	1	0.41	1.14	1 (GAF)	0.68	10.68	1 (MyR III)	8.47	87.23
	2 (GAF)	3.52	9.79	2 (MyR II)	3.19	50.08	2	1.07	11.02
	3	11.03	30.69	3	0.76	11.93	3	0.17	1.75
	4	0.98	2.73	4	1.74	27.32			
	5 (MyR I)	14.76	41.07						
	6	2.26	6.29						
	7	2.98	8.29						
					Yield (mg)		y%		
**Total GA I recovered**					4.2		8.03		
**Total MyR I and II recovered**					26.42		50.52		

MyR I and II – myricitrin I and II subfraction; MPF - main polyphenols subfraction; GA - gallic acid subfraction; SNT - supernatant; nFr - subfraction number; Y% - yield in percentage; Rep -repetition; Qt. - quantity.

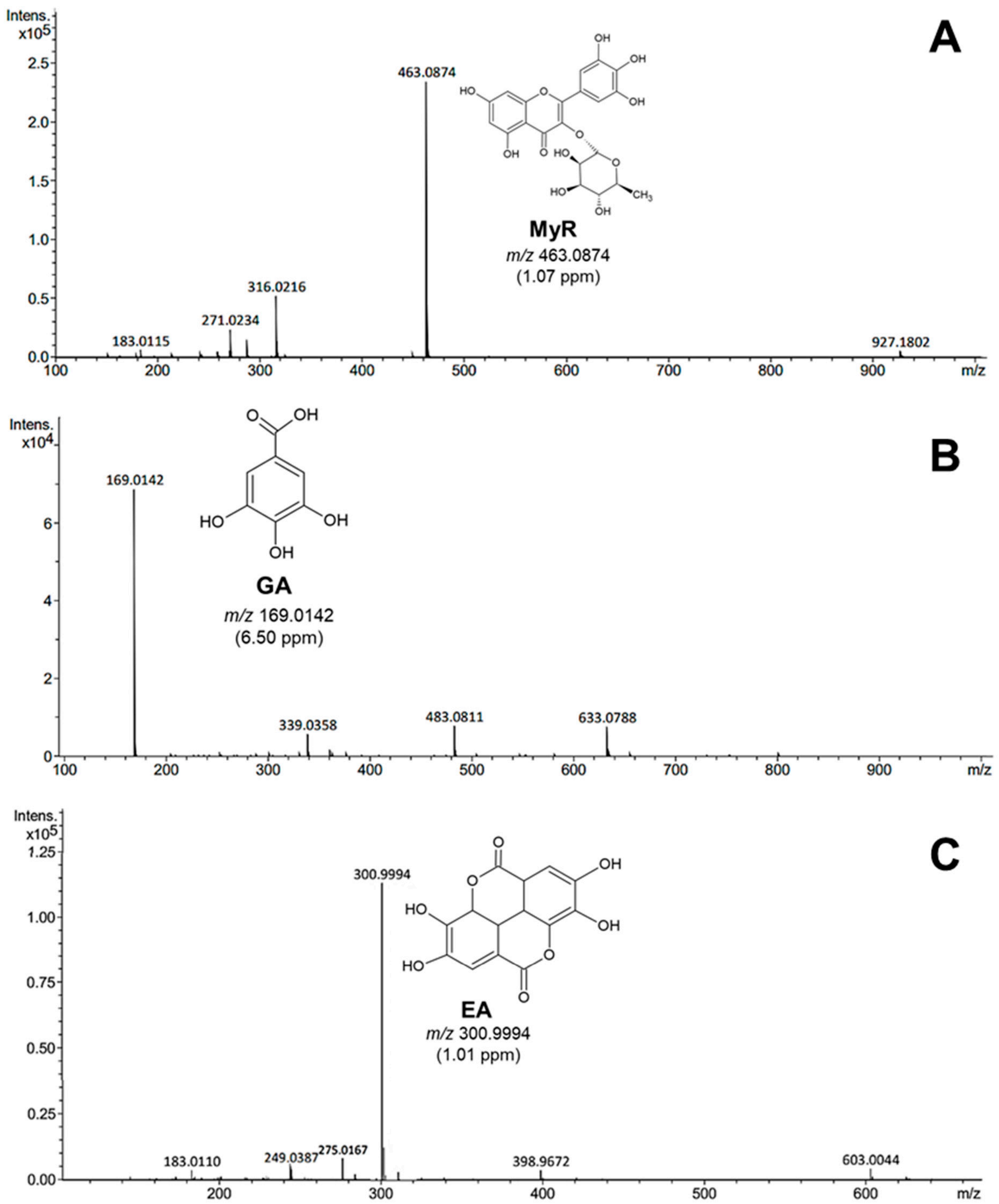

Figure. S10. Spectrometric profile of myricitrin (**A**), gallic acid (GA) (**B**) and ellagic acid (**C**) isolates from *E. uniflora* leaves evaluated by direct injection in ESI-HRMS/MS.
